# The role of offshore wind and solar PV resources in global low-carbon transition

**DOI:** 10.1126/sciadv.adx5580

**Published:** 2025-10-24

**Authors:** Yi Wen, Jiaxin Wu, Pengzhi Lin, Ying Min Low

**Affiliations:** ^1^ Centre for Offshore Research and Engineering, Department of Civil and Environmental Engineering, National University of Singapore, 1 Engineering Drive 2, Singapore 117576, Singapore.; ^2^ Ocean College, Zhejiang University, Zhoushan 316021, Zhejiang, China.; ^3^ Department of Mechanical Engineering, National University of Singapore, 9 Engineering Drive 1, Singapore 117575, Singapore.; ^4^ State Key Laboratory of Hydraulics and Mountain River Engineering, Sichuan University, 24, South Section No.1, Yihuan Road, Chengdu 610065, P. R. China.

## Abstract

With challenges such as land availability and regulatory constraints, offshore renewable energy sector is poised to play a pivotal role in the transition to a low-carbon future. Among offshore technologies, wind and solar photovoltaic (PV) have emerged as the most promising solutions. However, a global assessment of offshore resources, particularly solar PV, remains lacking. Hence, we identify suitable areas for offshore wind and solar PV development on the basis of economic feasibility, technical constraints, and environmental considerations and quantify the national potential for electricity production and CO_2_ reduction contributions. With a conservative assumption of using 1% of suitable areas, offshore wind and solar PV could generate ~6049 and 14,173 terawatt-hours of electricity annually. This would cover nearly 30% of the expected global electricity demand in 2050. The resulting reductions in carbon dioxide emissions could exceed 9 billion tonnes annually. These findings highlight the critical role offshore renewable energy can play in achieving a low-carbon future.

## Teaser

Offshore wind and solar PV could meet nearly 30% of 2050 electricity demand using only 1% of suitable ocean areas.

## INTRODUCTION

As carbon emissions and the impacts of climate change escalate, a growing number of countries have committed to achieving carbon neutrality, spurring the search for clean energy solutions to facilitate a low-carbon transition within the energy sector (*1*). Wind and solar photovoltaic (PV) are reshaping the global electricity supply as key drivers of the clean energy transition (*2*, *3*). In 2022, global wind and solar PV power generation reached ~3421.81 terawatt-hours (TWh), meeting around 12% of the electricity demand (*4*). According to the World Energy Outlook 2023, wind and solar PV are expected to meet nearly 70% of global electricity demand by 2050 under the Net Zero Emissions scenario (*5*).

Wind and solar PV systems are classified into onshore and offshore categories, depending on their installation environments. Although the installed capacity of onshore wind and solar PV systems has steadily increased, challenges related to land availability and regulatory constraints have emerged as substantial barriers to the widespread development of these technologies (*6*). In contrast, offshore wind and solar PV systems offer several advantages, such as avoiding land-use conflicts and minimizing visual impacts (*7*, *8*). In addition, offshore wind turbines benefit from stronger and more consistent wind resources (*9*), whereas offshore solar PV systems gain efficiency due to the water’s cooling effect (*10*), leading to enhanced power generation compared to their onshore counterparts. These unique advantages have driven the rapid expansion of offshore renewable energy projects globally, with offshore wind energy seeing particularly notable success. Now, most global offshore wind capacity is concentrated in the North Sea and neighboring regions of the Northwest Pacific and Atlantic Oceans (*11*). In 2022, offshore wind contributed nearly 30% of global wind power generation (*5*). However, these figures are expected to shift in the near future. Building on this momentum, advancements in floating PV technology are propelling the development of offshore solar PV resource, positioning them as a promising complement to offshore wind energy (*7*).

With over 50% of the world’s population residing within 100 km of the coastline (*12*) and ongoing advancements in offshore wind and solar PV technologies, there has been a noticeable shift toward using these vast ocean areas for the development of offshore wind and solar PV energy farms. Consequently, offshore wind and solar PV have the potential to serve as key drivers in accelerating decarbonization efforts and achieving global clean energy targets. Investigations on global offshore wind resources primarily focus on resource availability, seasonal characteristics, and optimal development locations [e.g., refs. (*13*–*15*)], whereas research on offshore PV resources is still in its early stages. Few studies have quantified the global potential of offshore solar PV resources, an area that researchers have explored far less than offshore wind energy. In addition, researchers have given limited attention to assessing how integrating offshore wind and solar PV can contribute to decarbonization in coastal nations. To address this gap, we investigate the decarbonization potential of offshore wind and solar PV resources by analyzing climate and ocean conditions, estimating available development areas, quantifying potential electricity generation, and evaluating their complementarity at national or regional scales. Bridging this knowledge gap is crucial for stakeholders as it provides the insights needed to make informed decisions regarding optimal locations and the scale of deployment necessary for offshore wind and solar PV to effectively contribute to the transition toward a decarbonized economy.

## RESULTS

### Global distribution of offshore wind and solar PV resources

**Fig. 1.**
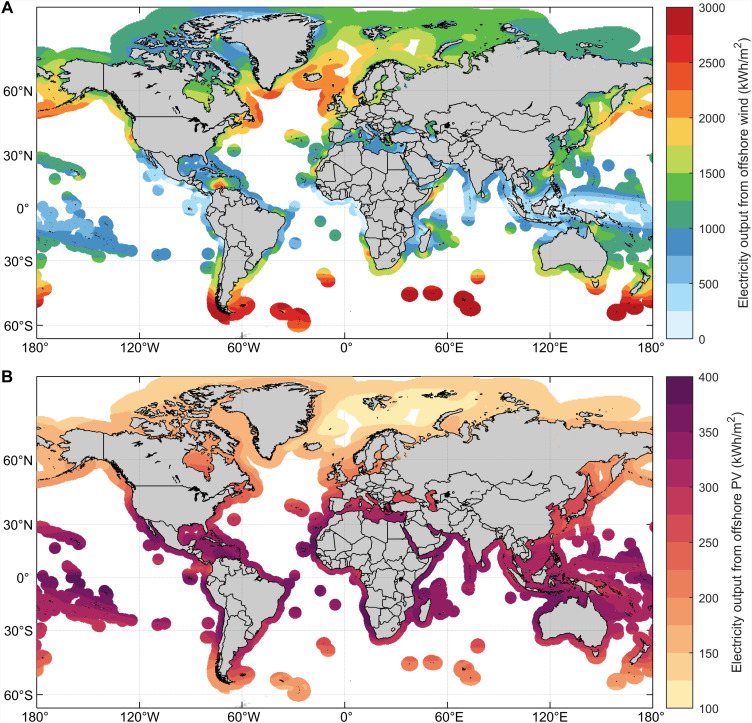
Global distribution of potential annual electricity output per unit area from offshore wind and solar PV. Estimated annual electricity output per unit area from (**A**) offshore wind and (**B**) solar PV within EEZs. The estimates are based on ERA5 wind speed at 100 m height and solar irradiance data from 2004 to 2023, accounting for the conversion efficiencies of offshore wind turbines and solar PV modules.

We begin by examining the spatial patterns of annual electricity output per unit area from offshore wind and solar PV. This analysis focuses exclusively on offshore wind and solar PV resources within marine exclusive economic zones (EEZs), which delineate the marine areas where coastal nations or regions have jurisdiction over both living and nonliving resources (*16*). Wind speed at 100 m height and solar irradiation data are obtained from the ERA5 dataset, i.e., the fifth-generation atmospheric reanalysis produced by the ECMWF (*17*). Figure 1 (A and B) displays the global distribution of potential annual electricity output per unit area from offshore wind and solar PV, respectively. There is a distinct variation in power output across different areas for both resources, primarily driven by differences in resource availability due to variations in latitude and topography. For offshore wind resources, because of Earth’s rotation, air flows primarily from east to west rather than from north to south, resulting in notable variations in wind speed distribution across different latitudes (*18*). Regions near the equator experience the weakest wind conditions due to the natural equatorial doldrums (*19*). In contrast, mid-latitude regions around 40° to 50° latitude, famously known as the Roaring Forties (*18*), benefit from notably stronger winds. Notable examples include seas near southern South America, southeastern Australia, southern New Zealand, Alaska, southeastern Canada, and northwestern Europe, where annual electricity output can exceed 2000 kilowatt-hours (kWh)/m^2^. In addition, specific straits and capes, such as the Taiwan Strait and northern South America, also exhibit relatively abundant offshore wind resources due to their unique topographical features.

Offshore solar PV resources display a spatial distribution opposite to that of offshore wind, with solar potential intensifying as latitude decreases. The most abundant PV resources are found in the region between the Tropic of Cancer and Tropic of Capricorn (approximately 23.45°N to 23.45°S), where solar irradiance is highest due to the relative motion of Earth and the Sun (*20*). In terms of resource density, regions such as North Africa, the Middle East, northern South America, South Asia, and northern Australia are considered golden areas for offshore solar PV development. These areas boast potential annual power generation of ~400 kWh/m^2^, making them highly advantageous for harnessing solar energy. However, it is important to note that offshore solar PV resources are influenced not only by latitude but also by weather factors such as cloud cover, which can notably affect solar energy availability (*21*).

Although our primary analysis is based on long-term average electricity output, interannual variability of offshore wind and solar PV resources has important implications for system reliability and planning. Additional analysis (fig. S1) shows that regions with high interannual variability in offshore wind output [coefficient of variation (CoV) > 25%] are mainly located in the tropical zones of Asia, the Pacific Ocean, and Africa. Offshore solar PV exhibits a similar pattern, with higher variability generally found in areas with lower average irradiance, except for localized peaks near the equatorial western Pacific. Overall, key offshore wind development regions typically show low variability (CoV < 10%), and solar PV tends to exhibit even lower interannual variability across most regions. These results suggest that offshore PV could play a stabilizing role in hybrid systems, complementing wind resources in regions with higher wind variability. This has important implications for long-term energy planning under climate variability.

### Areas of interest for global offshore wind and solar PV development

**Fig. 2.**
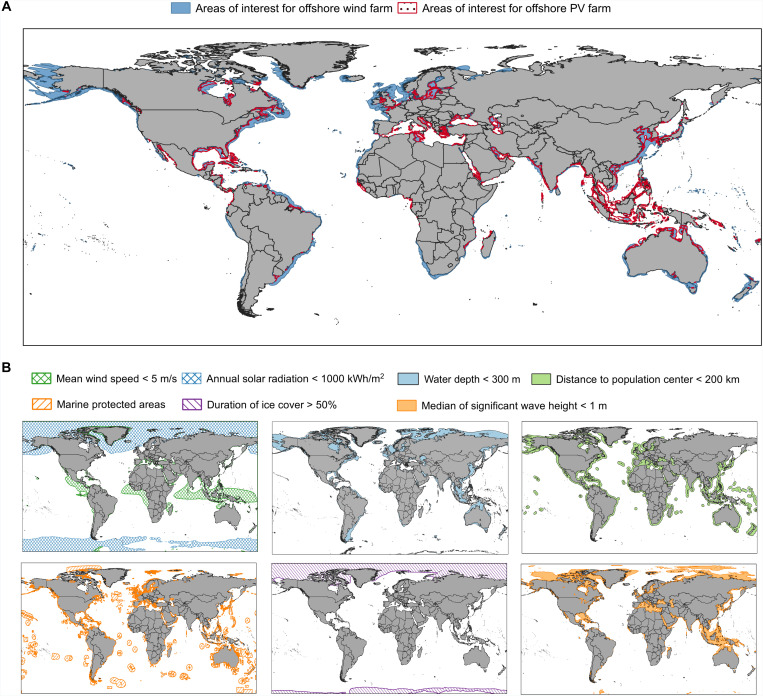
Areas of interest for offshore wind and solar PV farm development. (**A**) Locations of suitable areas for offshore wind and solar PV farms. (**B**) Specific suitability factors for offshore wind and solar PV development, including water depths less than 300 m, median significant wave height below 1 m (applicable to offshore PV farms only), and distance to population center less than 200 km, exclusion of marine protected areas, regions with ice cover duration larger than 50%, and areas that fail to meet minimum resource thresholds, i.e., annual average wind speeds below 5 m/s for wind resource and annual solar radiation below 1000 kWh/m^2^ for solar resource. Median significant wave height, minimum resource thresholds, and duration of ice cover data are derived from ERA5 data (1994 to 2023).

Although much of the world is surrounded by oceans, areas suitable for offshore wind and solar PV development are limited due to economic feasibility, technical constraints, and environmental considerations. To define the areas of interest for offshore wind and solar PV farms, the following key constraints apply: (i) areas must be within EEZs; (ii) water depths should be less than 300 m; (iii) distance to population centers should be within 200 km; (iv) marine protected areas are excluded; (v) regions with ice cover for more than 50% of the year are omitted; and (vi) areas that fail to meet minimum resource thresholds, i.e., annual average wind speeds below 5 m/s for wind resource and annual solar radiation below 1000 kWh/m^2^ for solar resource, are excluded. Specifically for offshore PV farms, because of their sensitivity to wave action, areas with a median significant wave height exceeding 1 m are excluded (Materials and Methods). Although floating solar PV farms have experienced remarkable growth, i.e., expanding at an annual rate of 133% over the past decade across more than 60 countries, most installations are still on inland reservoirs or lakes (*22*). Deploying floating PV in ocean environments presents unique challenges due to harsh marine conditions, particularly with respect to wave activity and extreme weather events (*23*) (fig. S2). Figure 2 presents the areas of interest for offshore wind and solar PV farm development, showing that only nearly 2.96 and 1.29% of global sea areas are suitable for offshore wind and solar PV development, respectively, based on the abovementioned criterion. A substantial portion of these suitable areas is concentrated in specific countries, including large regions in the United States (1,625,422 km^2^ for offshore wind and 317,580 km^2^ for offshore PV), Canada (1,192,120 km^2^ for offshore wind and 262,976 km^2^ for offshore PV), and Australia (1,113,664 km^2^ for offshore wind and 510,413 km^2^ for offshore PV) (data S1). It is important to note that the suitability estimates presented here are based on physical and environmental constraints and assume the availability of future floating platform technologies for offshore wind and solar PV, which are still under development and not yet cost-effective for widespread deployment.

### Electricity generation and CO_2_ emission reduction from offshore wind and solar PV

Our estimates suggest that offshore wind and solar PV could notably contribute to global energy needs and economic decarbonization efforts. Assuming that 1% of suitable areas are developed for offshore wind and solar PV, these resources could generate ~6049 and 14,173 TWh of electricity annually. This corresponds to 21 and 49% of the global electricity demand in 2022 (28,844 TWh) (*4*), nearly 9 and 22% of electricity demand by 2050 (~65,000 TWh) under the Net Zero Emissions scenario (*5*).

**Fig. 3.**
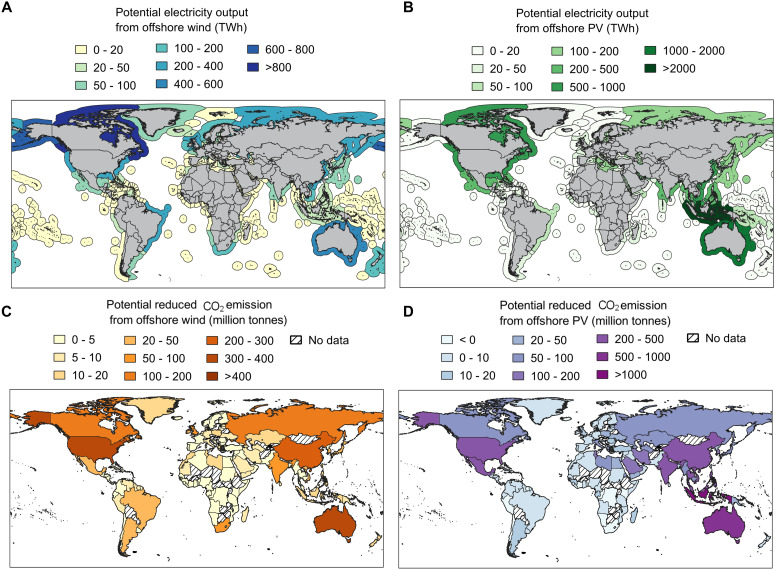
Potential electricity output and CO_2_ reduction from offshore wind and solar PV. (**A** and **B**) Estimated potential electricity output (TWh) from offshore wind (A) and solar PV (B) in various EEZs, assuming that 1% of areas of interest are used. (**C** and **D**) Estimated potential CO_2_ reduction (million tonnes) at national or regional scales from offshore wind (C) and solar PV (D), based on electricity generation from coastal EEZs.

To further understand the potential at national and regional levels, we estimated the electricity generation from offshore wind and solar PV within the areas of interest in each EEZ (Fig. 3, A and B) and assessed their contributions to reduce CO_2_ emissions for adjacent coastal countries and regions (Fig. 3, C and D, and fig. S3). The United States, with the largest area of interest, has the highest offshore wind potential, estimated at 995 TWh annually, which could reduce CO_2_ emissions by over 372 million tonnes. Canada follows closely, with an annual generation potential of 847 TWh, contributing to a reduction of 127 million tonnes of CO_2_ emissions. Australia, Russia, and China also demonstrate considerable potential, with each country able to achieve over 100 million tonnes of annual CO_2_ reductions through offshore wind alone. As a global leader in offshore wind energy, China has the potential to reduce its CO_2_ emissions by ~4% under the 1% area allocation scenario; however, this does not fully reflect offshore wind’s transformative potential in China’s energy strategy. If installations are expanded to 10% of suitable areas, offshore wind energy could reduce China’s CO_2_ emissions by up to 40%, highlighting its substantial decarbonization capacity. In total, for the coastal countries included in this study (~31% of global coastal nations), offshore wind energy could make a notable contribution (over 50%) to economic decarbonization efforts (data S1).

For offshore solar PV resources, tropical countries demonstrate the highest potential due to abundant sunlight exposure. Indonesia, for example, has the greatest offshore solar PV generation potential at 2153 TWh/year, potentially reducing CO_2_ emissions by over 1.35 billion tonnes—equivalent to six times the nation’s 2022 emissions. Australia ranks second, with an offshore solar PV potential of 1761 TWh annually, which could lead to a reduction of 920 million tonnes of CO_2_. China is showcasing the feasibility of large-scale offshore PV deployment. Despite a relatively modest reduction potential (~9% of national emissions), China’s sustained investment in floating PV technology establishes it as a notable contributor to global decarbonization efforts. Overall, for the coastal countries assessed in this study (covering around 27% of total coastal nations), offshore solar PV resources alone could make considerable strides (over 50%) toward economic decarbonization goals (data S1). It is worth noting that, in countries where the carbon intensity of electricity is already very low (e.g., Albania, ~24 gCO_2_ kWh^−1^), the development of offshore PV could paradoxically lead to a negative impact, i.e., potentially increasing total CO_2_ emissions due to the life cycle emissions associated with PV system production and installation.

### Complementarity of offshore wind and solar PV

Individual offshore wind and solar PV systems face resource variability, leading to imbalances between power generation and grid load (*24*). To address this, many offshore solar PV projects are now codeveloped with offshore wind farms to reduce costs and enhance grid stability (*24*). Now, several wind and PV joint development pilot projects are under demonstration operation. For instance, the 0.5-megawatt (MW) Haiyang project in Shandong, China (*25*), and the 5-MW OFS project on the west coast of the Netherlands (*26*). In addition, a grid-scale 540-MW hybrid wind-solar project in Italy has been initiated through a collaboration agreement between SolarDuck, Green Arrow Capital, and New Developments s.r.l. (*27*). This project represents a milestone in advancing offshore floating solar PV systems and highlights the immense potential of this technology in Italy and across the broader Mediterranean region (*27*).

**Fig. 4.**
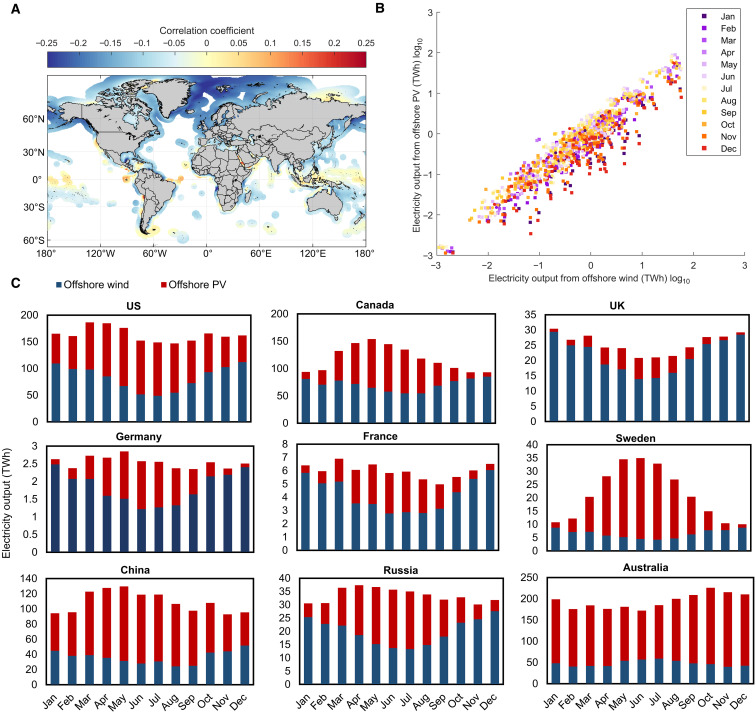
Complementarity of electricity output from offshore wind and solar PV resources. (**A**) Pearson correlation coefficient between hourly electricity outputs from offshore wind and solar PV at each grid unit within EEZs, covering the period of 1994 to 2023. (**B**) Scatterplot showing the potential monthly electricity outputs (TWh) from offshore wind and solar PV across various countries or regions. The circles represent countries or regions in the Southern Hemisphere, whereas the squares represent those in the Northern Hemisphere. For a fair assessment, offshore wind and solar PV outputs are normalized to equal annual electricity production. (**C**) Comparison of potential electricity output by month, highlighting seasonal complementarity between offshore wind and solar PV resources in selected countries.

Offshore solar and wind energy are complementary to some extent both spatially and temporally as their nature attributes these two resources (*28*). Spatially, offshore wind power tends to be more suitable for countries at mid-to-high latitudes, whereas tropical regions benefit more from offshore solar PV (Fig. 1). Temporally, there is often an inverse relationship between solar irradiance and wind strength: During the day, strong sunlight coincides with weaker winds, whereas at night, reduced sunlight aligns with stronger winds due to larger temperature differences. Mathematically, the Pearson correlation coefficient is used to quantify this temporal complementarity, with low or negative values indicating high complementarity as they suggest that the peaks in the two resources do not coincide (*29*). Regions with high complementarity include the North Sea and Baltic Sea (e.g., UK, Germany, and Denmark), the North Atlantic (US East Coast), and the North Pacific [e.g., Alaska (USA), China, and Russia] (Fig. 4A). On a seasonal scale, offshore solar and wind energy resources also exhibit complementary patterns. In the Northern Hemisphere, offshore wind resources peak in January and December when offshore solar PV output is weakest, whereas in June and July, offshore solar PV potential is at its highest and offshore wind resources are at their lowest (figs. S4 and S5). Hence, in most countries or regions in the Northern Hemisphere, offshore wind power complements offshore solar PV generation primarily in December and January, whereas solar PV generation supports wind power predominantly in June and July (Fig. 4B). Conversely, in the Southern Hemisphere, this complementarity pattern is reversed, with solar PV generation complementing wind power during the winter months and wind power supporting solar PV during the summer months (Fig. 4B and figs. S4 and S5). Figure 4C illustrates the monthly comparison of potential electricity output from offshore wind and solar PV in selected countries, highlighting seasonal complementarity in countries such as Germany and UK. This monthly complementarity between offshore wind and solar PV resources enables a more balanced and reliable power generation system.

## DISCUSSION

### Decarbonization potential from offshore wind and solar PV

This study aims to assess the decarbonization potential of global offshore renewable energy, with a focus on offshore wind and solar PV resources. Using nearly 20 years (1994 to 2023) of ERA5 meteorological data, we analyze the spatiotemporal distribution of offshore wind and solar PV resources, their potential electricity output and CO_2_ reduction capacity, and their complementarity. The findings provide valuable insights that could inform national and regional strategies for offshore wind and solar PV deployment. First, offshore wind and solar PV resources exhibit notable spatial and temporal heterogeneity, necessitating tailored deployment strategies based on regional resource availability and geographical characteristics. For example, southern South America is more suitable for offshore wind energy development, whereas tropical nations such as Indonesia should prioritize offshore solar PV. Second, although only 2.96 and 1.29% of global sea areas are deemed suitable for offshore wind and solar PV deployment, their electricity generation potential remains substantial. Even under a conservative scenario using only 1% of suitable areas, offshore wind and solar PV could exceed current electricity demand in certain countries and regions. For instance, Denmark’s offshore wind potential is 1.5 times its current electricity demand, whereas Malaysia’s offshore solar PV potential is 3.4 times its demand. On a global scale, offshore wind and solar PV could contribute notably to the global energy supply and decarbonization efforts, generating ~6049 and 14,173 TWh annually, which would account for 9 and 22% of projected global electricity demand by 2050 under the Net Zero Emissions scenario. It should be noted that the main results are based on current technological constraints. However, any change in the assumed thresholds could substantially affect the outcomes. For example, if offshore deployment is extended to water depths up to 1000 m in the future, as technological advancements make this feasible, the potential electricity output from offshore wind and solar PV could increase notably. Under this scenario, they could contribute ~12 and 27% of the projected global electricity demand in 2050, respectively. This expanded deployment would also result in CO_2_ emission reductions of ~2.28 and 7.14 billion tonnes, respectively (data S2). Third, in regions such as the North Sea and Baltic Sea (e.g., UK, Germany, Denmark), the North Atlantic (e.g., US East Coast), and the North Pacific (e.g., Alaska, China, and Russia), offshore wind and solar PV resources exhibit high complementarity. Coastal European nations such as Germany and Italy could benefit notably from integrated offshore wind and solar PV development, a strategy already being pursued in ongoing North Sea projects.

### Economic feasibility

Although this study primarily assesses the technical potential of offshore wind and solar PV on the basis of resource availability, it is important to recognize that the actual deployment of these technologies is strongly influenced by economic feasibility, particularly the levelized cost of electricity (LCOE). The LCOE is a comprehensive metric reflecting all life cycle costs—capital expenditures (CAPEX), operational expenditures (OPEX), and maintenance—amortized over the total electricity output (*30*). Offshore wind technologies, although more costly than onshore systems due to logistical and environmental challenges, have undergone considerable cost reductions over the past decade. From 2011 to 2023, the global average installed cost declined by 55% to $2800/kW, whereas operation and maintenance (O&M) costs fell to around $0.022/kWh in China and $0.023 to $0.030/kWh in Europe, driven by greater competition, operational experience, and turbine innovations (*31*). Despite higher upfront costs, the typically higher capacity factors of offshore wind help reduce the levelized cost per unit of electricity. As a result, the global average LCOE dropped by 63%, from $0.203/kWh in 2010 to $0.075/kWh in 2023 (*31*). These reductions reinforce the importance of our resource assessment: Key areas identified in our study, such as northern Europe and coastal China, not only exhibit strong wind potential but also benefit from maturing markets and a lower LCOE, exemplified by Denmark at $0.048/kWh in 2023 (*31*).

In contrast, offshore solar PV remains an emerging technology, and its economic feasibility is not yet well established due to limited large-scale deployments and a lack of long-term cost data. Offshore floating solar PV still has inevitably high costs due to the loads from waves requiring reinforcement and small-scale development. The LCOE of pilot offshore floating PV projects is estimated at approximately $0.382/kWh (*32*). This is more than five times the average LCOE of offshore wind. However, as the offshore technology is currently at research and development stage with only small pilots installed to date, it is expected that we will see notable cost reductions as projects scale. At present, offshore solar PV power generation tends to be deployed together with offshore wind power [e.g., (*33*, *34*)]. As offshore wind expands, the space between turbines offers opportunities to deploy floating solar PV arrays. Shared grid and storage infrastructure, along with synergies in labor, vessels, and maintenance, can reduce OPEX (*35*). These efficiencies may lower the overall LCOE of hybrid systems to levels comparable to, or even below, standalone offshore wind or land-based solar. For example, in the Netherlands, the LCOE of offshore solar is projected to fall to €50/MWh ($0.054/kWh) by 2030 and €40/MWh ($0.043/kWh) by 2050 (*32*). As such, the large technical potential identified in our resource assessment may become increasingly accessible in the coming decades, provided that supportive policies, technological improvements, and integrated offshore energy system planning continue to reduce costs and enable hybrid deployments of wind and PV.

### Future challenges

Despite the growing maturity of offshore wind deployment in regions such as Northern Europe, several technical challenges remain, particularly as future projects extend into deeper waters and more complex ocean environments (*36*). Now, most offshore wind turbines are installed on fixed-bottom foundations in shallow coastal zones (*11*). However, for water depths beyond 50 to 60 m, such as in parts of the Mediterranean or East Asia, floating offshore wind is considered the most economically viable solution. This transition necessitates the development of robust floating structures and mooring systems, capable of withstanding extreme marine conditions. Similarly, floating offshore PV systems must be designed to survive harsh wave climates, which remains an engineering and economic challenge (*37*).

The lack of reliable ocean meteorological data also imposes a major constraint, especially during early-stage site assessment and risk analysis in deep-sea regions (*38*). The high cost of offshore measurements often poses a barrier to project development by limiting the availability of accurate wind, wave, and irradiance data in underexplored offshore areas (*39*).

The environmental impacts associated with large-scale offshore renewable installations are still not fully characterized. Offshore wind turbine foundations may alter local sea creatures, hydrodynamic processes, and sediment transport (*40*), whereas the coverage of floating PV systems could potentially affect biogeochemical cycles, nutrient exchange, and marine ecosystem dynamics, especially in stratified or eutrophic regions (*41*). These environmental uncertainties should be further addressed in future work to ensure the sustainable deployment of offshore technologies.

Grid integration and energy system compatibility pose additional challenges for large-scale offshore renewable deployment. The variable and weather-dependent nature of offshore wind and solar PV poses reliability issues for power systems with limited flexibility or weak infrastructure (*42*). Mitigation strategies include the deployment of storage systems, advanced forecasting methods, demand-side management, and hybrid system design that leverages the temporal complementarity between wind and solar resources (*42*). Furthermore, the development of offshore transmission infrastructure, such as substations, subsea cables, and grid interconnections, poses logistical and economic challenges, especially for installations located in remote or deep-sea areas.

### Limitations

Despite these promising findings, this study has several limitations. First, the analysis relies on ERA5 meteorological data, which provide a reliable global-scale estimation of offshore energy potential. However, for countries or regions with limited maritime areas, the spatial resolution may be insufficient to accurately capture local offshore energy potential. For instance, Singapore’s estimated offshore solar PV potential is reported as zero, which does not reflect its actual feasibility, as offshore PV projects have already been deployed there. Second, the estimated resource potential is highly sensitive to constraint thresholds, meaning that adjustments to these parameters could notably alter the results. To improve the accuracy of local and regional offshore energy assessments, we encourage refining these thresholds or incorporating additional constraints, such as economic limitations due to adverse marine conditions. Third, although this study demonstrates the temporal complementarity between offshore wind and solar PV, the practical benefits of codevelopment depend on various factors, including technology selection, installation ratios, energy storage integration, and electricity demand variations across different timescales. Fourth, although offshore wind and solar PV represent promising low-carbon energy solutions, their effective deployment depends on resource intensity, electricity demand, grid infrastructure, financial investment, and technological expertise. These aspects should be further explored in future research to enhance the feasibility and scalability of offshore renewable energy systems.

## MATERIALS AND METHODS

### Electricity output from offshore wind and solar PV

Power output from offshore wind primarily depends on the instantaneous wind speed and the performance characteristics of offshore wind turbine. Key parameters influencing turbine performance include the cut-in, rated, and cut-out wind speed, the rotor’s swept area, and the power conversion efficiency. Wind speeds below cut-in speed or above cut-out speed do not contribute to energy generation, whereas power output reaches a maximum and remains constant between rated speed and cut-out speed. The instantaneous power output from a wind turbine can be expressed as follows

$$P_{\text{wind}}=\left\{\begin{array}{cc} 0 & U<U_{\text{in}}\\ \frac{1}{2}\rho \cdot C_{p}\cdot A_{T}\cdot U^{3} & U_{\text{in}}\leq U<U_{r}\\ P_{e} & U\geq U_{\text{out}}\\ 0 & U<U_{\text{in}} \end{array}\right.$$ (1)

where ρ represents the air density, set to 1.213 kg/m^3^, reflecting the average condition commonly used in global offshore wind resource assessments (*7*). *U*_in_, *U*_r_, and *U*_out_ denote the cut-in, rated, and cut-out wind speed, respectively. These are defined as 4, 13.5, and 25 m/s, based on typical specifications of large-scale offshore wind turbines, consistent with previous studies and industry reports (*43*, *44*). *A*_T_ refers to the rotor’s swept area, a critical factor that determines the amount of wind energy captured. It is set to 31,400 m^2^, corresponding to a rotor diameter of 200 m, representative of next-generation offshore wind turbines. *C*_p_ is the power coefficient, which reflects the efficiency of converting wind kinetic energy into electrical power, typically ranges between 0.28 and 0.42 for offshore turbines (*43*). We adopt a median value of 0.32, consistent with the global average used in large-scale wind potential assessments, providing a balanced and conservative estimate across diverse regions and turbine designs. *P*_e_ denotes the rated power of the turbine. For modeling purposes, it corresponds to the electrical output achieved when wind speed reaches the rated speed, beyond which turbines cease operation to avoid structural damage.

Offshore solar PV energy generation, on the other hand, is estimated on the basis of the plane-of-array (POA) irradiance, the surface area, and temperature-adjusted conversion efficiency of the PV module. The instantaneous power output of the PV module is given by the equation (*45*)

$$P_{\text{PV}}=I_{\text{POA}}\cdot \eta \cdot A_{S}$$ (2)

where *I*_POA_ denotes the POA solar irradiance, and *A*_S_ presents the surface area of the PV module. η indicates the conversion efficiency of the PV module. In most applications, *I*_POA_ is derived from global horizontal irradiance (GHI) using decomposition and transposition models that account for module tilt and orientation (*46*). However, for offshore floating PV modules installed horizontally (i.e., with a tilt angle of 0°), the *I*_POA_ can be reasonably approximated by GHI (*47*, *48*). In this study, we used the ERA5 surface solar radiation downwards (SSRD) variable, which represents the accumulated shortwave solar radiation (in J/m^2^) on a horizontal surface, and it can be converted GHI (in W/m^2^) by dividing by the accumulation period (3600 s for hourly data) and directly used it as *I*_POA_ for offshore PV systems. The conversion efficiency depends on the temperature of the PV module, which can be estimated as follows (*45*, *49*)

$$\eta =\eta_{\text{ref}}\left[1-\beta \left(T_{c}-T_{\text{ref}}\right)\right]$$ (3)

In this equation, $\eta_{\text{ref}}$ is the electrical efficiency of the PV module under standard conditions, set at 0.17, with the reference temperature (*T*_ref_) of 25°C (*44*). β = 0.005°C^−1^ is the temperature coefficient, representing the efficiency drop per unit increase in temperature. This value is widely reported for the commercial PV module (*44*). *T*_c_ denotes the operating temperature of the PV module, which can be estimated on the basis of ambient conditions and wind speed using the empirical model developed for floating PV systems (*7*)

$$T_{c}=c_{1}+c_{2}\cdot T_{a}+c_{3}\cdot I-c_{4}\cdot V_{w}$$ (4)

where *T*_a_ is the ambient air temperature, and *V*_w_ is the wind speed at 10 m height, both obtained from ERA5. *c*_1_, *c*_2_, *c*_3_, and *c*_4_ are empirical constants with values of 2.0458, 0.9458, 0.0215, and −1.2376, respectively; these values were derived from studies on the thermal performance of floating PV systems (*7*).

If the swept area of wind turbine blades or the surface area of PV modules is not taken into account, the power output per unit area can be calculated for both. After calculating the instantaneous power output from offshore wind and solar PV, we derived the 20-year hourly mean power output at each grid point. The annual electricity generation (*E*_mean_) is then estimated by multiplying these hourly mean power output (*P*_mean,hourly_) by the total number of hours in a year (i.e., 8760 hours), as shown below

$$E_{\text{mean}}=P_{\text{mean},\text{hourly}}\times 8760$$ (5)

This approach provides an estimate of yearly energy generation per unit area, without assuming a specific turbine swept area or PV module size.

Interannual variability of offshore renewable energy resources is quantified using the *CoV* of annual electricity output from offshore wind and solar PV. The *CoV* is defined as the ratio of the SD ( $\sigma_{E}$ ) to the annual electricity generation (*E*_mean_), expressed as follows

$$\text{CoV}=\frac{\sigma_{E}}{E_{\text{mean}}}$$ (6)

### Identification of areas of interest

When identifying suitable sea areas for the deployment of offshore wind and PV technologies, several key factors must be considered: EEZs, minimum resource threshold, water depth, marine protected areas, ice cover duration, distance to population center, and wave height (applicable only to offshore PV). This study focuses on the development of offshore wind and PV resources within EEZs, i.e., areas where nations or regions hold exclusive rights to exploit marine resources in adjacent waters (*16*). To ensure the economic feasibility of offshore renewable energy projects, minimum resource thresholds are applied. For wind power projects, sea areas with an annual average wind speed below 5 m/s are excluded as such areas are unlikely to support viable wind energy generation (*50*). For offshore PV, regions with annual total radiation less than 1000 kWh/m^2^ are excluded (*51*). Although there is no widely accepted threshold for minimum radiation levels for solar PV systems, most studies suggest that an annual total radiation of 1200 to 1500 kWh/m^2^ is considered acceptable (*52*, *53*). However, in areas like Europe and Japan, where radiation levels are lower (~1000 to 1200 kWh/m^2^ per year), PV remain economically viable due to high electricity prices and supportive subsidy policies. Drawing on successful offshore wind farm projects and anticipated advancements in technology, we assume that offshore wind turbines and floating PV systems are generally installed in areas with water depths less than 300 m. Now, Norway hosts the deepest offshore wind project, with installations at depths ranging from 260 to 300 m (*54*). In the future, floating wind turbines are anticipated to be deployed at depths of up to 1000 m, with ambitious plans already underway in countries such as the United States and Japan (*55*, *56*). Furthermore, the development of marine resources must avoid marine protected areas (*57*). The presence of ice can notably affect the buoyancy and structural loads of floating systems, affecting both the installation and operation of offshore wind and PV projects. As such, this study limits areas with ice cover duration to less than 50% (*21*). Only sea areas within 200 km of regions with a population density exceeding 300 people/km^2^ are considered (*58*). This is because countries with higher population densities often face land constraints for onshore wind and solar projects, making offshore renewable energy development a more viable and attractive alternative (fig. S6). For offshore PV, exposure to severe storm waves can cause substantial displacement and structural damage of floating panels, negatively affecting energy generation (*37*). Thus, the median significant wave height for offshore PV installations is restricted to less than 1 m based on a previous study (*7*). Using these criteria, we use QGIS tools to delineate suitable polygonal areas under each constraint, creating a comprehensive spatial map of potential development sites (Fig. 2).

It is important to note that the criteria applied in this study are primarily based on project experience and extensive consultations with stakeholders. Despite these standardized criteria, the global suitability of offshore wind and PV projects is ultimately dependent on local conditions, including technological capacity, national policy frameworks, economic viability, marine environmental impacts, and community acceptance. These factors play a decisive role in determining the feasibility and long-term success of offshore wind and PV projects.

### Potential reduced CO_2_ emission on national or regional scales

We first calculate the potential electricity output from offshore wind and solar PV within area of interest in each EEZ, assuming that 1% of the area of interest is designated for development. Each grid cell area (0.05° × 0.05°) is calculated using longitudinal and latitudinal dimensions. The grid cell length at latitude is constant at ~5.56 km, whereas the grid cell length at longitude varies with latitude, reaching its maximum at the equator. For offshore wind farms, we adopt a standard grid layout, with a spacing of 5 rotor diameters (5 m by 200 m in this work) in the main wind direction and 3 rotor diameters between turbines in the perpendicular direction, as suggested by the GASP project (*59*). For offshore solar PV farms, continuous installation of PV modules without spacing intervals on the sea surface is adopted. On the basis of this approach, the number of wind turbines and the coverage area for PV modules within each grid cell are estimated, allowing electricity generation at each grid cell to be calculated. Using the area of interest within each country or region’s EEZ and the power output from wind turbines and PV modules, which can be calculated via Eqs. 1 and 2, national or regional estimates for electricity generation from offshore wind and solar PV are derived.

The potential CO_2_ emission reduction from offshore wind and solar PV is estimated for each country or region by multiplying the electricity output from these sources by the respective carbon intensity (fig. S7) and adjusting for life cycle CO_2_-equivalent (CO_2_e) emissions (*60*), as expressed below

$$∆{\text{CO}}_{2}=\text{AEP}\times \left(\text{CI}-\text{LCE}\right)$$ (7)

where $∆{\text{CO}}_{2}$ indicates the potential annual CO_2_ emission reduction, and *AEP* is the total annual electricity generation from offshore wind or solar PV, which can be calculated by multiplying *E*_mean_ in Eq. 5 by the available area. *CI* represents the carbon intensity of national or regional electricity, which quantifies the CO_2_ emissions released per unit of electricity generated. The carbon intensity data for different countries were sourced from ref. (*4*) (fig. S2), using 2022 data to ensure consistency across national metrics. *LCE* represents the life cycle CO_2_ emissions of offshore wind or solar PV systems. This metric accounts for total greenhouse gas emissions generated over the entire life span of the system, including raw material extraction, manufacturing, transportation, installation, O&M, and decommissioning. For offshore wind, life cycle CO_2_ emissions are reported to range from 8 to 35 gCO_2_/kWh, according to the IPCC (*60*). In this study, we adopt a conservative median value (~12 gCO_2_/kWh) within this range to reflect the current state of industrial practice and the technological maturity of large-scale offshore wind development. Because offshore PV technology is still under development and lacks a definitive carbon intensity value, we use the IPCC’s life cycle CO_2_e emissions estimate for utility-scale PV systems, which is ~48 gCO_2_/kWh (*60*). It should be noted that the average carbon intensity of a country or region may underestimate or overestimate mitigation in regions where marginal electricity generation comes from sources with notably different emissions profiles. In addition, we calculate the potential reduction proportion of CO_2_ emissions for each country or region, defined as the potential CO_2_ emission reduction from offshore wind and PV divided by the country or region’s total annual CO_2_ emissions (fig. S3). Total CO_2_ emissions are estimated by multiplying each country or region’s electricity demand (fig. S8) by its carbon intensity.

### Complementarity of electricity output from offshore wind and solar PV

Pearson correlation coefficient is used to assess the complementarity of offshore wind and solar PV resources. Areas where the correlation between these resources is negative are more attractive as this indicates that their respective generation peaks do not coincide and thus can reduce power variability (*61*). Pearson correlation coefficient (*R*) can be calculated as follows (*62*)

$$R=\frac{1}{N}\sum_{i=1}^{N}\frac{\left(P_{\text{wind},i}-{\bar{P}}_{\text{wind}}\right)\left(P_{\text{PV},i}-{\bar{P}}_{\text{PV}}\right)}{\sigma_{P_{\text{wind}}}\sigma_{P_{\text{PV}}}}$$ (8)

where ${\bar{P}}_{\text{wind}}$ and ${\bar{P}}_{\mathrm{PV}}$ are the mean values of instantaneous power from wind (*P*_wind,i_) and PV (*P*_PV,i_), respectively; $\sigma_{P_{\text{wind}}}$ and $\sigma_{P_{\mathrm{PV}}}$ are the SD of *P*_wind,i_ and *P*_PV,i_, respectively; and *N* is time step number of the hourly offshore wind and solar PV.

For a fair assessment of national or regional complementarity on a month scale, offshore wind and solar PV outputs are normalized to equal annual electricity production. If one energy source generates more electricity annually than the other, a normalization factor is applied to the higher output. This factor is calculated as the ratio of the smaller annual electricity production to the larger annual electricity production, ensuring balanced comparison and evaluation of their contributions.
