# Supplementary material for: The role of offshore wind and solar PV resources in global low-carbon transition
Source: Sci Adv. 2025 Oct 24;11(43):eadx5580. doi: 10.1126/sciadv.adx5580 (PMC12551713; doi:10.1126/sciadv.adx5580)
Supplement: Supplementary file 1 — Figs. S1 to S8 Legends for data S1 and S2 [file sciadv.adx5580_sm.pdf]

Science Advances
AAAS

Supplementary Materials for

The role of offshore wind and solar PV resources in global low-carbon transition

Yi Wen *et al.*

Corresponding author: Pengzhi Lin, cvelinpz@scu.edu.cn; Ying Min Low, ceelowym@nus.edu.sg

Sci. Adv. **11**, eadx5580 (2025)
DOI: 10.1126/sciadv.adx5580

The PDF file includes:

Figs. S1 to S8
Legends for data S1 and S2

Other Supplementary Material for this manuscript includes the following:

Data S1 and S2

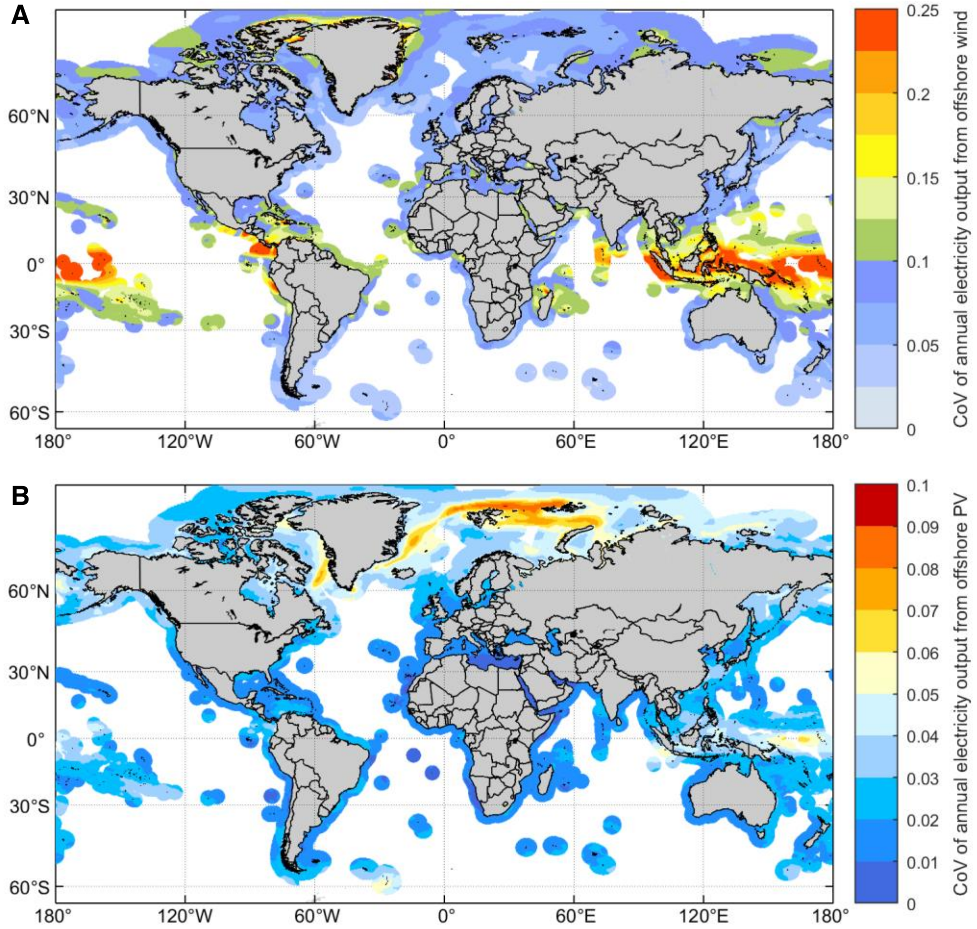

Fig. S1. Global distribution of interannual variability of electricity output from offshore wind and PV. CoV of annual electricity output from (A) offshore wind and (B) solar PV within EEZs. The estimates are based on wind speed at 100 m height and solar irradiance data from ERA over the period from 2004 to 2023.

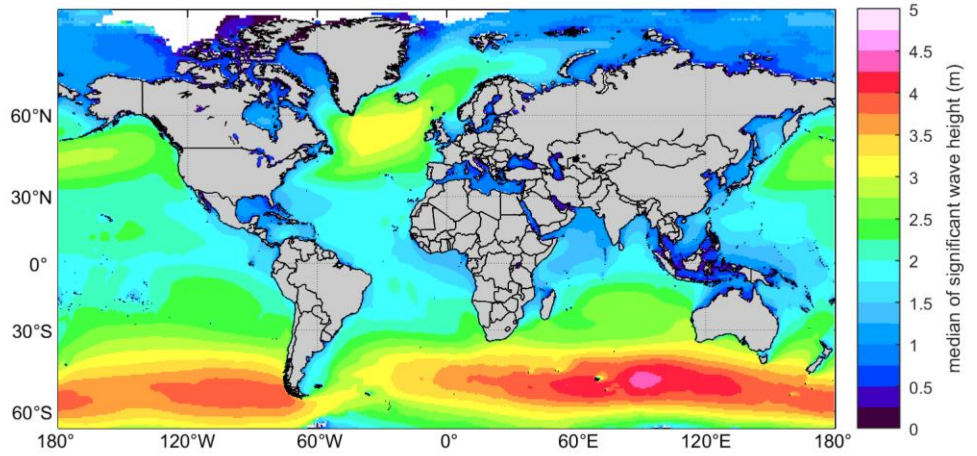

Fig. S2. Global distribution of median significant wave height. The calculation is based on significant wave height from ERA over the period from 2004 to 2023.

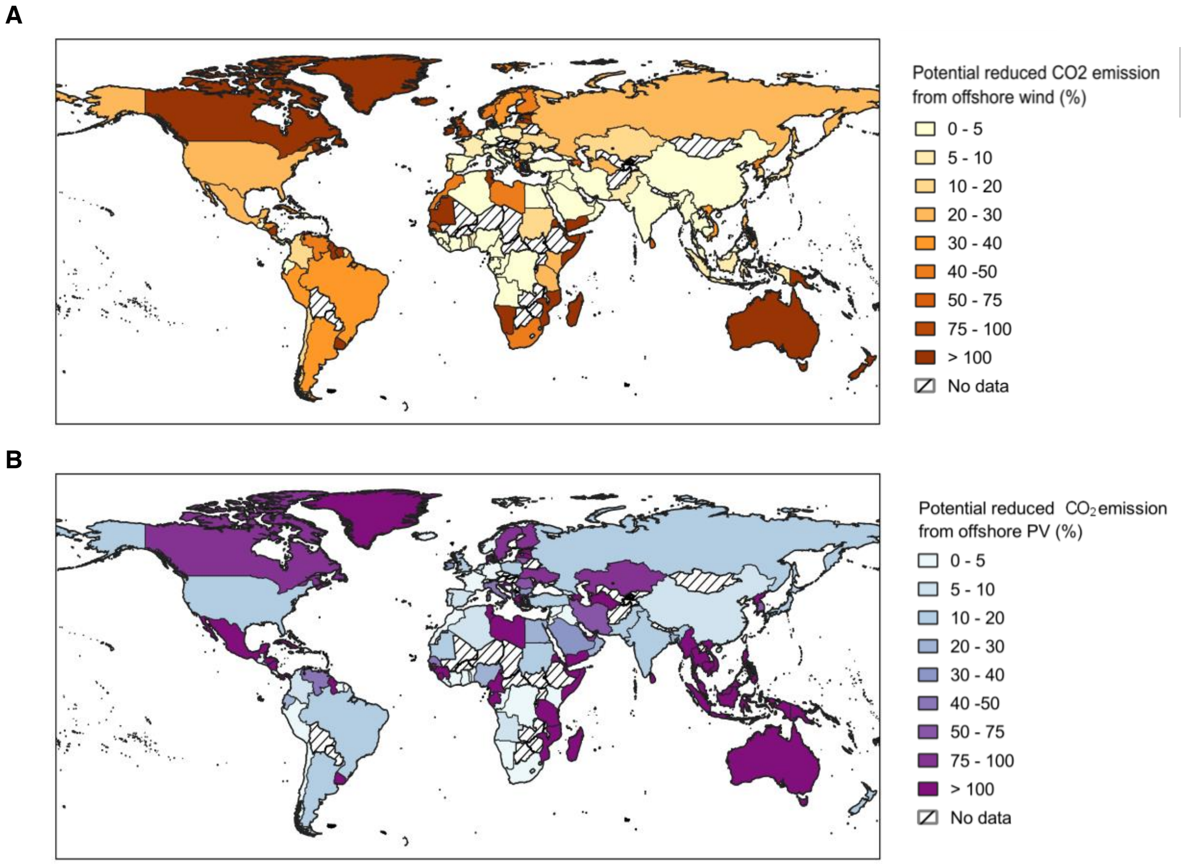

Fig S3. Potential CO_2 reduction from offshore wind and solar PV. Estimated potential CO_2 reduction (%) at national or regional scales from offshore wind (A) and solar PV (B), based on electricity generation from coastal EEZs. 1% of interest sea areas are utilized.

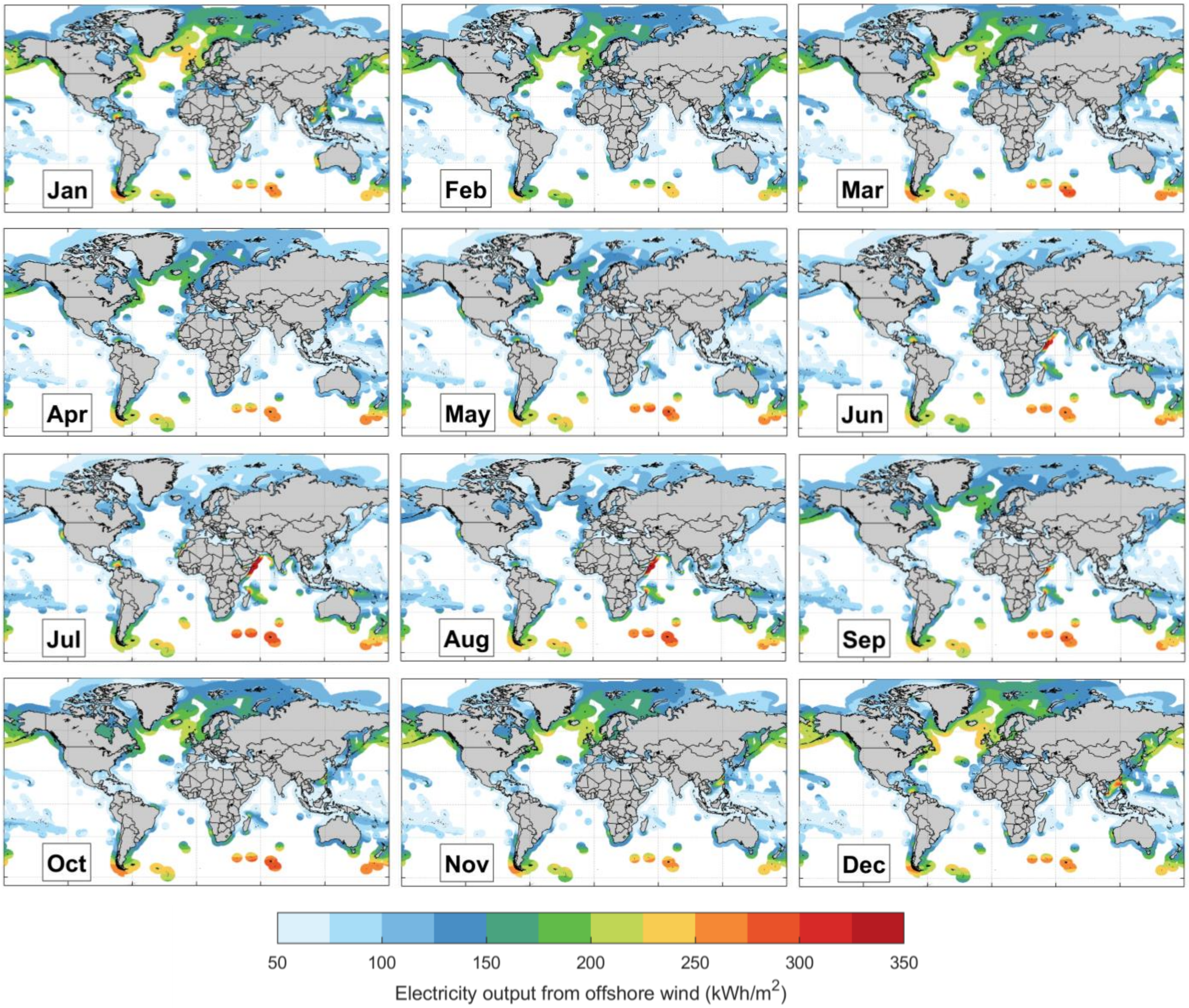

Fig S4. Seasonal distribution of potential electricity output per unit area from offshore wind in EEZs. The estimates are based on ERA wind speed at 100 m height from 2004 to 2023, accounting for the conversion efficiencies of offshore wind turbines.

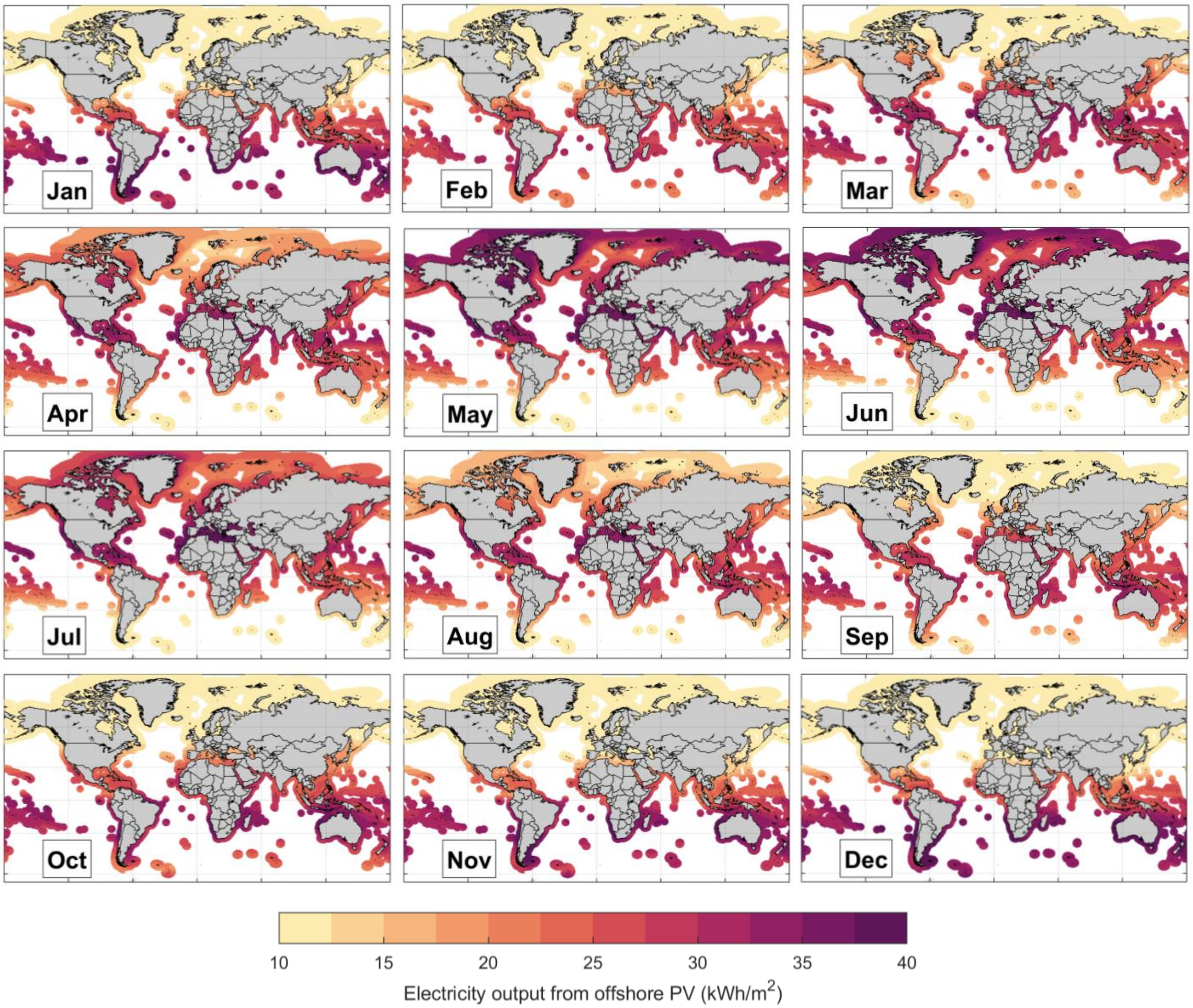

Fig S5. Seasonal distribution of potential electricity output per unit area from offshore solar PV in EEZs. The estimates are based on ERA solar irradiance data from 2004 to 2023, accounting for the conversion efficiencies of solar PV modules.

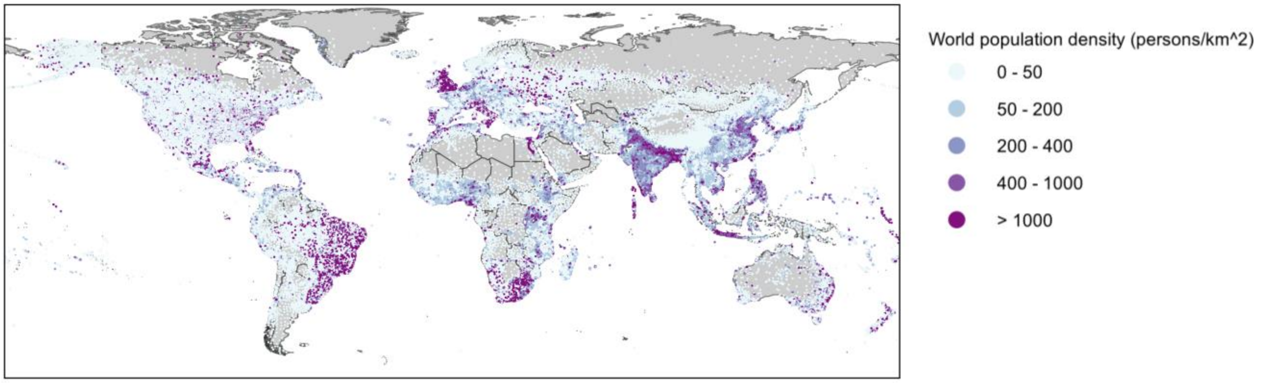

Fig S6. World population density.

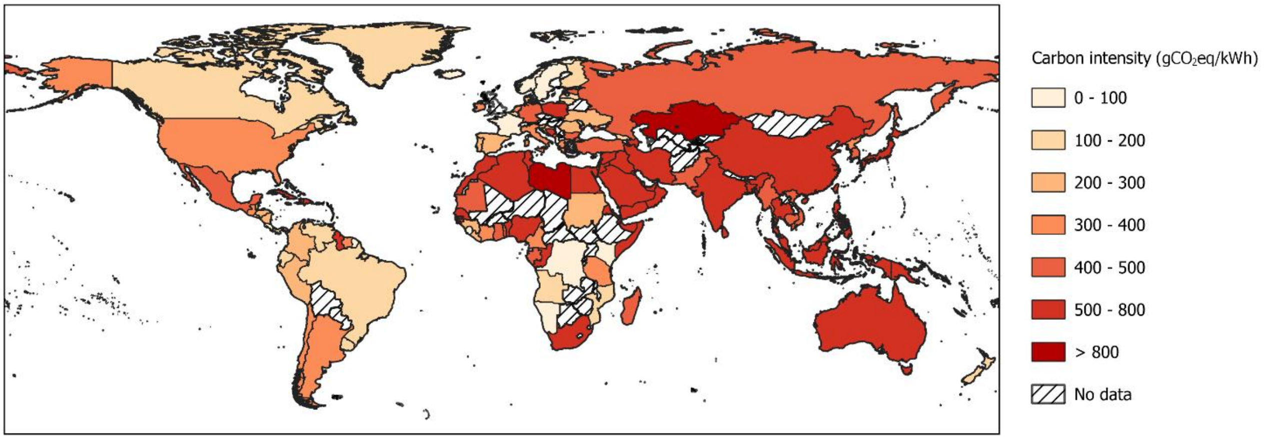

Fig S7. Carbon intensity (gCO_2eq/kWh) of electricity in 2022. Only coastal countries or regions with EEZ are shown.

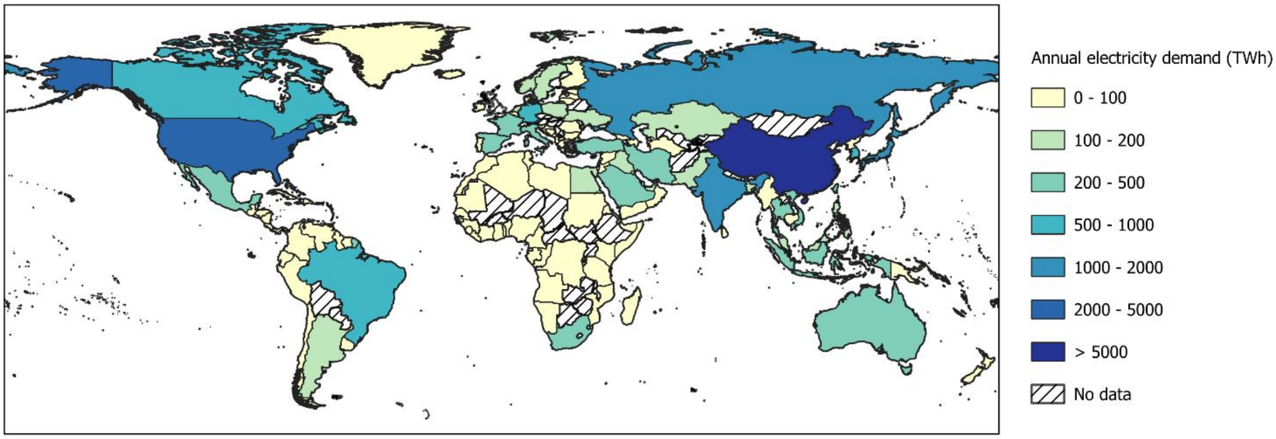

Fig S8. Annual electricity demand (TWh) in 2022. Only coastal countries or regions with EEZ are shown.

Data S1. Summary of national or regional offshore wind and solar PV electricity generation potential and CO_2 reduction by EEZs, under constraints including water depth ⩽300 m and additional technical and environmental criteria as described in the Materials & Methods.

Data S2. Summary of national or regional offshore wind and solar PV electricity generation potential and CO_2 reduction by EEZs, under constraints including water depth ⩽1000 m and additional technical and environmental criteria as described in the Materials & Methods.
